# Target site as the main mechanism of resistance to imazamox in a *Euphorbia heterophylla* biotype

**DOI:** 10.1038/s41598-019-51682-z

**Published:** 2019-10-28

**Authors:** Antonia M. Rojano-Delgado, João M. Portugal, Candelario Palma-Bautista, Ricardo Alcántara-de la Cruz, Joel Torra, Esteban Alcántara, Rafael De Prado

**Affiliations:** 1 Department of Agricultural Chemistry and Edaphology, University of Córdoba, Córdoba, Spain; 2 Department of Biosciences, Valoriza-Research Center for Endogenous Resources Valorization, Polytechnic Institute of Beja, Beja, Portugal; 3 Department of Chemistry, Federal University of São Carlos, São Carlos, Brazil; 4 Department d’Hortofructicultura, Botànica i Jardineria, Agrotecnio, Universitat de Lleida, Lleida, Spain; 5 Department of Agronomy, University of Córdoba, Córdoba, Spain

**Keywords:** Plant ecology, Plant evolution, Abiotic

## Abstract

*Euphorbia heterophylla* is a weed species that invades extensive crop areas in subtropical regions of Brazil. This species was previously controlled by imazamox, but the continuous use of this herbicide has selected for resistant biotypes. Two biotypes of *E. heterophylla* from southern Brazil, one resistant (R) and one susceptible (S) to imazamox, were compared. The resistance of the R biotype was confirmed by dose-response assays since it required 1250.2 g ai ha^−1^ to reduce the fresh weight by 50% *versus* 7.4 g ai ha^−1^ for the S biotype. The acetolactate synthase (ALS) enzyme activity was studied using ALS-inhibiting herbicides from five different chemical families. The R biotype required the highest concentrations to reduce this enzyme activity by 50%. A Ser653Asn mutation was found in the ALS gene of the R biotype. The experiments carried out showed that imazamox absorption and metabolism were not involved in resistance. However, greater ^14^C-imazamox root exudation was found in the R biotype (~70% of the total absorbed imazamox). Target site mutation in the ALS gene is the principal mechanism that explains the imazamox resistance of the R biotype, but root exudation seems to also contribute to the resistance of this biotype.

## Introduction

*Euphorbia heterophylla* L. is a dicotyledonous weed belonging to the *Euphorbiaceae* family. The species originated in the tropical and subtropical regions of America, where most of the affected crop areas are located^1–3^. Until the 1990s, the presence of this species in cotton, soybean and corn fields was fairly well controlled with acetolactate synthase-inhibiting herbicides (ALS-inhibiting herbicides) (HRAC group B, WSSA group 2). However, due to poor control, the invasion range of *E. heterophylla* has increased to include more crop areas^4–6^, other countries such as Mexico and the USA^7,8^ and even other continents such as Europe^9^, causing great economic losses. This lack of control is due to the evolution of new *E. heterophylla* biotypes resistant to these herbicides^6,10^. The first known case of resistance to ALS-inhibiting herbicides in this species was reported in Brazil (1993) and some years later in Paraguay (1995)^6^. Since then, other *E. heterophylla* cases with ALS-inhibiting herbicide resistance (including imazamox) have been found in large areas of Brazil (2004), also selecting for resistance to herbicides with other modes of action (MOA)^11–14^.

Imazamox [(5-(methoxymethyl)-2-(4-methyl-5-oxo-4-propan-2-yl-1H-imidazol-2-yl) pyridine-3-carboxylic acid)] belongs to the chemical family of imidazolinones within the ALS-inhibiting herbicides. It is a systemic herbicide that acts in early post-emergence stages, causing the inhibition of the ALS enzyme (EC 2.2.1.6), which is involved in the synthesis of the essential branched-chain amino acids isoleucine, leucine and valine^15^.

To study the basis of herbicide resistance, all the mechanisms should be considered. These mechanisms can be classified as target-site resistance (TSR) and non-target-site resistance (NTSR) mechanisms, depending on whether the target protein is involved or not, respectively^16,17^. Currently, imazamox resistance is explained by the appearance of point mutations in the ALS gene (TSR mechanism)^18–20^, the lack of herbicide absorption and translocation^21,22^ and the herbicide metabolism^22–24^ (all these have NTSR mechanisms) in different grass and broadleaf weeds with resistance to ALS-inhibiting herbicides.

Several point mutations are the most frequent mechanisms of resistance to imazamox found in the cases studied across weed species^24–27^. Eight point mutations (Ala122, Pro197, Ala205, Asp 376, Arg377, Trp574, Ser653 and Asn654) have been well described^28,29^, and these mutations show differential cross-resistance patterns to the different chemical families of ALS-inhibiting herbicides. Although TSR mechanisms usually provide high levels of herbicide resistance, some NTSR mechanisms can also provide high levels^16,17^. In fact, several NTSR mechanisms (alone or together with TSR mechanisms) can influence the resistance level within a single plant.

These NTSR mechanisms can differ depending on the species and MOA. Studies of herbicides with different MOAs^16,17,30,31^ revealed that variations in the pattern of herbicide absorption and translocation can also provide high resistance levels because they can reduce the herbicide concentration in meristematic tissues to non-toxic levels. Differential herbicide translocation may be caused by different factors, such as the herbicide being retained/sequestered, herbicide metabolism and its metabolites translocating inside the plant^32^, or large amounts of herbicide being translocated and quickly exuded via the root system, as postulated in the only known case for MCPA in a *Raphanus raphanistrum* L. biotype^33^.

The main objective of this work was to study in depth the basis of the high imazamox resistance of one *E. heterophylla* biotype from Brazil compared to the low resistance of one susceptible biotype of this species, analysing all the possible resistance mechanisms involved, both TSR and NTSR. This research represents the first attempt to unravel the resistance mechanisms to ALS-inhibiting herbicides in this species.

## Results

### Dose-response assays

The imazamox dose needed to reduce the fresh weight (ED_50_) by 50% in the R biotype plants was 1250.2 g ai ha^−1^ *versus* 7.4 g ai ha^−1^ for the S biotype (Table 1, Supplementary Fig. S1). These results obtained from the fresh weight showed that the R biotype was 168 times more resistant than the S biotype. Based on the dose to achieve 50% mortality (LD_50_), the R biotype was 116 times more resistant than the S biotype (Table 1, Supplementary Fig. S1). Considering that the recommended field dose is 40 g ai ha^−1^, the R biotype can survive more than 50 times this dose, making it impossible to control this biotype with imazamox.

**Table 1:** Parameters of the Log–Logistic equation ± standard error used to calculate the imazamox effective doses (g ai ha^−1^) required to reduce the fresh weight (ED_50_) and/or cause plant mortality (LD_50_) by 50% in two biotypes (S, susceptible; R, resistant) of *E. heterophylla*.

	Biotype	d	b	R^2^	*P* value	ED_50_ /LD_50_	RF
fresh weight reduction (ED)	S	99.9 ± 1.2	0.59 ± 0.02	0.98	<0.0001	7.4 ± 0.3	168.3
	R	100.2 ± 1.8	0.88 ± 0.03	0.99	<0.0001	1250.2 ± 48.2	
mortality (LD)	S	100.2 ± 0.9	2.36 ± 0.12	0.99	<0.0001	19.4 ± 0.4	116.3
	R	99.9 ± 0.7	3.15 ± 0.21	0.99	<0.0001	2253.1 ± 38.3	

^a^Y = d/1 + (x/g)^b^ where: *d* is the coefficient corresponding to the upper asymptote, *b* is the slope of the line, *x* the imazamox concentration, and *g* is the imazamox concentration at the inflection point, hence the ED_50_ or LD_50_. ^±^Standard error of the mean (*n* = 5). R^2^ aj = 1 − (sums of squares of the regression/corrected total sums of squares). *P* value = significance level of the nonlinear model. ^c^Resistance factors [RF = ED_50_ or LD_50_ (R)/ED_50_ or LD_50_ (S)].

### ALS enzyme activity assays

The *in vitro* activity of the ALS enzyme in the absence of herbicides was similar in the R and S biotypes (220.2 ± 9.2 and 213.4 ± 17.2 nmol of acetoin mg^−1^ protein h^−1^, respectively). This enzyme activity was reduced by 50% with 33.7 µM imazamox in the S biotype, while for the R biotype, 538.4 µM (approximately 16 times more herbicide) was necessary. This fact shows that the enzyme plays a very important role in the resistance to imazamox. For the rest of herbicides, the activity was also reduced, but the magnitude depended on the herbicide and biotype (Table 2). The I_50_ values for the S biotype were very low for bensulfuron and florasulam (<2 μM), indicating that these herbicides are able to stop ALS enzymatic activity for this biotype, while the I_50_ values for the R biotype were higher than for the S biotype. The RF (resistant factor) values for bensulfuron, bispyribac, florasulam and flucarbazone in the R biotype were 12, 2, 525 and 17, respectively.

**Table 2:** Parameters of the log–logistic equations^a^ used to calculate the concentration (µM) of the ALS-inhibiting herbicides needed to inhibit the ALS activity by 50% (I_50_) in two biotypes (S, susceptible; R, resistant) of *E. heterophylla*.

Herbicide^b^	Biotype	c	d	b	R^2^aj	*P* value	I_50_	RF
Bensulfuron (SU)	S^†^	—	100.0 ± 5.1	2.05 ± 0.11	0.99	<0.0001	1.5 ± 0.1	12.5
	R	7.1 ± 0.3	100.8 ± 7.3	1.36 ± 0.08	0.99	<0.0001	19.1 ± 0.7	
Bispyribac (PTB)	S	10.4 ± 0.5	101.0 ± 3.1	0.99 ± 0.05	0.98	<0.0001	137.7 ± 6.7	2.0
	R	21.9 ± 1.1	100.1 ± 4.9	2.31 ± 0.10	0.99	<0.0001	269.3 ± 6.4	
Florasulam (TP)	S^†^	—	101.5 ± 3.9	1.18 ± 0.04	0.99	<0.0001	1.3 ± 0.1	524.7
	R	16.9 ± 0.7	100.8 ± 1.2	0.93 ± 0.04	0.98	<0.0001	692.6 ± 11.1	
Flucarbazone (SCT)	S	3.9 ± 0.2	100.2 ± 6.8	0.80 ± 0.02	0.99	<0.0001	20.7 ± 1.0	17.3
	R	12.5 ± 0.6	100.2 ± 2.6	1.28 ± 0.04	0.99	<0.0001	358.3 ± 9.5	
Imazamox (IMI)	S	1.8 ± 0.1	100.0 ± 1.9	0.56 ± 0.03	0.98	<0.0001	33.7 ± 1.0	16.0
	R	19.6 ± 1.0	100.2 ± 3.1	0.17 ± 0.01	0.99	<0.0001	538.4 ± 8.1	

^a^*Y* = *c* + {(*d* − *c*)/[1 + (*x*/*g*)^*b*^]} (four parameters) where: *c* and *d* are the coefficient corresponding to the lower and upper asymptotes, respectively; *b* is the slope of the line, *x* the herbicide concentration, and *g* is the herbicide concentration at the inflection point, hence the I_50_. ^†^Regression analyses adjusted to a model of three-parameters (Y = d/1 + (x/g)) assuming that the lower limit is zero. ^b^ALS chemical classes: sulfonylureas (SU), imidazolinones (IMI), triazolopyrimidines (TP), pyrimidinylthiobenzoates (PTB) and sulfonylamino-carbonyl-triazolinones (SCT). ±Standard error of the mean (*n* = 5). R^2^aj = 1 − (sums of squares of the regression/corrected total sums of squares). *P* value = significance level of the nonlinear model. RF = Resistance factor = I_50_R/I_50_S.

### ALS sequencing

**Figure 1:**
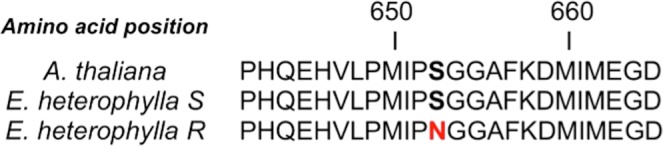
Partial alignment of protein sequences of the ALS gene in ALS-susceptible and ALS-resistant *E. heterophylla* biotypes. The red color indicates a change at the position 653 from Ser (S) to asparagine (N).

The potential mutation sites known to confer resistance to ALS inhibitors in the ALS gene sequences were amplified in the R and S *E. heterophylla* biotypes. An amino acid substitution from serine to asparagine was found at position 653 in the ALS gene of the R biotype (Fig. 1).

### Foliar retention of imazamox

The R and S *E. heterophylla* biotypes did not show differences in leaf area or shoot weight (data not presented), but the amount of imazamox solution retained was higher in the R biotype (379.4 ± 28.3 µL g^−1^ dry weight) than in the S biotype (256.3 ± 32.2 µL g^−1^ dry weight).

### Absorption, translocation, root exudation and visualization of ^14^C-imazamox applied via foliage

In this assay, one drop (1 µL) of ^14^C-imazamox was applied to one leaf, and after 3 hours, more than 90% of the ^14^C-imazamox applied was inside the plants of both biotypes, with no differences between them (Table 3). At this time, more than 80% of the absorbed ^14^C was located in the treated leaf in both biotypes (Table 3). Translocation from the treated leaf to the rest of the plant increased with time; at 96 HAT, only 23% and 7% of the ^14^C absorbed remained in the treated leaf for the S and R biotypes, respectively. The images obtained with the phosphor imager at 96 HAT confirmed the higher translocation in the R biotype (Supplementary Fig. S2). However, lower contents were found in the root than in the shoot. The amount of ^14^C in roots increased until 24 HAT, similar to that in both biotypes, and then decreased until 96 HAT more markedly in the R biotype. At 24 HAT, an increase in the amount of ^14^C exudated into the nutrient solution was found, reaching at 96 HAT higher values in the R biotype (65%) than in the S biotype (38%) (Table 3).

**Table 3:** Absorption and translocation percentage (%Trans.) of ^14^C-imazamox (from the total absorbed) from 3 to 96 hours after treatment in two *E. heterophylla* biotypes (S, susceptible; R, resistant) grown in a hydroponic systems.

%Trans.	Biotype	Hours after treatment					
		3	6	12	24	48	96
Absorption (% total applied)	S	92.4 ± 4.2^Aa^	94.3 ± 3.3^Aa^	98.3 ± 3.0^Aa^	98.9 ± 2.3^Aa^	98.9 ± 2.6^Aa^	98.9 ± 2.0^Aa^
	R	93.7 ± 3.2^Aa^	95.2 ± 2.6^Aa^	98.2 ± 3.1^Aa^	98.6 ± 3.0^Aa^	98.8 ± 3.0^Aa^	98.9 ± 3.1^Aa^
Treated leaf	S	88.5 ± 2.7^Aa^	84.7 ± 3.1^ABa^	70.8 ± 4.9^Ca^	45.0 ± 3.2^Ea^	22.0 ± 1.6^Fdef^	22.8 ± 3.4^Fc^
	R	84.0 ± 2.7^ABa^	78.0 ± 1.6^Bb^	53.6 ± 1.5^Db^	45.4 ± 2.2^Ea^	19.6 ± 2.7^Ffg^	7.0 ± 1.0^Ge^
Shoots	S	6.5 ± 1.2^Eb^	7.7 ± 1.4^Ede^	11.4 ± 3.0^De^	25.5 ± 3.0^ABb^	29.5 ± 5.4^Abcd^	22.6 ± 1.7^Bc^
	R	9.8 ± 2.2^DEb^	12.0 ± 2.1^Dc^	21.3 ± 2.3^Bc^	22.6 ± 3.8^Bbc^	22.5 ± 1.4^Bdef^	15.6 ± 0.7^Cd^
Roots	S	3.1 ± 2.5^Gcd^	5.6 ± 1.8^FGe^	15.7 ± 1.8^BCDd^	23.6 ± 0.8^Abc^	20.4 ± 3.7^ABefg^	14.6 ± 1.7^CDd^
	R	4.2 ± 1.2^FGc^	7.9 ± 1.6^EFde^	21.9 ± 3.9^ABc^	24.0 ± 3.8^Abc^	16.8 ± 1.4^BCg^	7.3 ± 0.5^EFe^
Exuded	S	1.9 ± 0.1^GHd^	1.8 ± 0.3^GHf^	1.9 ± 0.3^GHf^	5.7 ± 0.5^Ee^	26.2 ± 1.9^Ccd^	37.6 ± 2.2^Bb^
	R	1.6 ± 0.3^Hd^	1.8 ± 0.4^GHf^	2.4 ± 0.5^FGf^	8.1 ± 0.2^Dd^	39.7 ± 2.2^Ba^	64.8 ± 5.5^Aa^

±Standard error of the mean (*n* = 3). Means followed by the same lowercase per column (translocation of ^14^C-imazamox within a time evaluated) or uppercase per double plant section row (translocation of ^14^C-imazamox between biotypes at different time intervals) does not differ by the Tukey test (P < 0.05).

### Imazamox metabolism

The NTSR mechanism involving herbicide metabolism was also investigated in these two biotypes. In this work, metabolites of imazamox were not found in either the biotype of *E. heterophylla* or in the nutrient solutions after herbicide foliar application (Table 4). This indicated that metabolism was not involved in the imazamox resistance of the R biotype and that the ^14^C detected inside the plant (in the previous experiment) can be ascribed to ^14^C-imazamox. However, this assay provides more information than just imazamox metabolism. The total amount of herbicide in the shoot, root and nutrient solution was quantified by LC from 6 to 168 HAT (Table 4), supporting the previous assay (absorption, translocation, root exudation and visualization of ^14^C-imazamox applied via foliage). The maximum values in the shoot were found at 6 HAT and gradually decreased until 168 HAT, being more pronounced in the R than in the S biotype. At 168 HAT, the amount of herbicide remaining in the shoot was 0.22 µmol for the S biotype and only 0.08 µmol in the R biotype. The amount of herbicide in roots increased until 24 HAT, in a similar way for both biotypes, and then it decreased specially in the R biotype, as occurred in the previous experiment. In the nutrient solution, there was a progressive increase in the herbicide amount with evaluation time that was more important in the R biotype than in the S biotype.

**Table 4:** Total amount of imazamox (expressed in µmol) and its metabolites (Mt) in two *E. heterophylla* biotypes (S, susceptible; R, resistant) at different times in shoot, root and nutrient solution of hydroponically grown plants.

Plant section	Biotype	Hours after treatment											
		6		12		24		48		96		168	
		Imazamox	Mt	Imazamox	Mt	Imazamox	Mt	Imazamox	Mt	Imazamox	Mt	Imazamox	Mt
Shoot	S	0.526 ± 0.028^a^	—	0.470 ± 0.036^a^	—	0.405 ± 0.015^a^	—	0.296 ± 0.001^a^	—	0.262 ± 0.001^b^	—	0.219 ± 0.001^c^	—
	R	0.503 ± 0.023^a^	—	0.426 ± 0.005^b^	—	0.388 ± 0.003^b^	—	0.240 ± 0.002^b^	—	0.129 ± 0.001^d^	—	0.083 ± 0.001^e^	—
Root	S	0.032 ± 0.002^c^	—	0.090 ± 0.006^d^	—	0.135 ± 0.002^c^	—	0.117 ± 0.002^d^	—	0.084 ± 0.001^e^	—	0.118 ± 0.002_d_	—
	R	0.044 ± 0.004^b^	—	0.124 ± 0.008^c^	—	0.137 ± 0.001^c^	—	0.096 ± 0.001^e^	—	0.042 ± 0.001 ^f^	—	0.033 ± 0.002 ^f^	—
Solution	S	0.010 ± 0.005^d^	—	0.011 ± 0.002^e^	—	0.033 ± 0.001^e^	—	0.093 ± 0.002^e^	—	0.217 ± 0.015^c^	—	0.245 ± 0.015^b^	—
	R	0.010 ± 0.003^d^	—	0.014 ± 0.003^e^	—	0.046 ± 0.001^d^	—	0.227 ± 0.015^bc^	—	0.371 ± 0.005^a^	—	0.460 ± 0.005^a^	–

±Standard error of the mean (n = 5). Means followed by the same letter per column does not differ by the Tukey test (P < 0.05).

### Accumulation, distribution and visualization of ^14^C-imazamox applied to the roots

In this assay, the exudation of ^14^C could not be evaluated because there was ^14^C-imazamox in the nutrient solution. No large differences in accumulation were found between the R and S biotypes, although accumulation was slightly greater in the R biotype (Table 5). Regarding the distribution, at 24 HAT, the herbicide content was more than twice as high in the shoot that in the roots, with the R and S biotypes having a similar distribution. However, at 96 HAT, the % of herbicide decreased in the shoot and increased in the root, making this change much more important in the R biotype. The visualization of ^14^C at 96 HAT confirmed the higher accumulation of ^14^C in the R biotype (Supplementary Fig. S3).

**Table 5:** Accumulation and distribution percentages of ^14^C-imazamox (of the total applied to the nutrient solution) by the roots of two *E. heterophylla* biotypes (S, susceptible; R, resistant) grown in a hydroponic systems at 24 and 96 HAT.

HAT	Biotype	Accumulation (% from total applied)	Distribution (% from accumulated)	
			Root	Shoot
24	S	11.7 ± 1.8a	24.5 ± 1.0b	78.5 ± 3.6a
	R	12.4 ± 1.1a	36.2 ± 2.8a	62.4 ± 2.8b
96	S	10.2 ± 0.9b	28.1 ± 1.5b	70.3 ± 1.6a
	R	15.5 ± 2.0a	75.6 ± 3.0a	23.5 ± 1.3b

±Standard error of the mean (n = 3). Different letters within each column and sampling time differ statistically by the Tukey test (P < 0.05).

## Discussion

The high RF value in the dose-response experiments with imazamox for the R *E. heterophylla* biotype suggested that a TSR mechanism (mutation) may be involved in the resistance to imazamox and that root exudation, a NTSR mechanism, could contribute to it, consistent with the findings of Breccia *et al*.^34^ and Ghanizadeh and Harrington^16,17^.

The presence of a TSR mechanism was demonstrated by enzymatic studies with different ALS-inhibiting herbicides. From the I_50_ values of the R biotype, it can be deduced that the most effective herbicide should be bensulfuron at the enzymatic level. However, differences in herbicide effectiveness at the plant or field level may vary, as other resistance mechanisms have not been considered. The low inhibition of ALS activity in the R biotype by all tested herbicides revealed cross resistance and suggested that a TSR mechanism, specifically the occurrence of mutation(s) in the ALS gene, may contribute to imazamox resistance since no significant differences in basal enzymatic activity were found with the S biotype; as a result, mechanisms such as differences in copy number or gene expression can be discarded as potential resistance mechanisms to imazamox^21,35,36^.

Mutations at different positions of the ALS active site have been found in R biotypes of several weed species^23,26,37^. Amino acid changes in positions that confer cross resistance to imidazolinones and sulfonylureas include Ala-205, Asp-376, Trp-574 and Ser-653. Furthermore, some of these mutations may be responsible for the patterns of cross resistance to other families of ALS inhibitors^38,39^. In this case, the R *E. heterophylla* plants presented a Ser653Asn mutation. Amino acid substitutions at Ser653 are more likely to influence IMI binding than SU binding because the first group of herbicides binds at a lower depth in the ALS protein active site than does SU^40^. Mutations occurring in this position (Ser653Thr) were previously described in *Amaranthus powellii* and *A. retroflexus* that conferred resistance only to IMI herbicides^41^. However, IMI-resistant *Setaria viridis* and *Bromus tectorum* populations carrying the Ser653Asn mutation presented cross resistance to the different families of ALS-inhibiting herbicides, with the highest level of IMI and low or moderate levels to the other families of ALS inhibitors^42,43^. In this study, the highest resistance according to ALS enzyme activity was to TRI, while the levels of resistance to IMI, SU, SUCAR and PYR were low. Therefore, it can be concluded that the Ser653Asn mutation found in the ALS gene of the R biotype may be responsible for the cross-resistance patterns observed in the enzyme activity.

Regarding the other resistance mechanisms, metabolism as a mechanism of NTSR to imazamox has been described in *Triticum aestivum*, where the main identified metabolites were imazamox-OH and imazamox-glucose^22,23,44^. This behaviour was also observed in other species, such as *Papaver rhoeas*^24^, where imazamox metabolism is involved in its resistance. However, in this work, imazamox metabolites were not found in R or S *E. heterophylla* plants or in the nutrient solution, indicating that metabolism was not involved in the imazamox resistance of the R biotype. The amounts of herbicide detected in the shoot, root and nutrient solution were proportional to those quantified in the foliar absorption and translocation assays. These results corroborated the hypothesis that the substance moving within both R and S plants corresponded to the herbicide.

Resistance or tolerance can also be related to differences in herbicide leaf retention, an NTSR mechanism, between different species^45–48^ and different biotypes^49–51^. In relation to herbicide retention capacity, the leaf area has also been related to ALS inhibitor resistance in a few cases. For example, in *Amaranthus powellii*, the biotype resistant to ALS-inhibiting herbicides (IMI and SU) produced a 58% smaller leaf area than did the S biotype^36^. However, in sulfonylurea-resistant *Kochia scoparia*, the R and S biotypes produced similar leaf areas and dry weights^35^. Seemingly, *E. heterophylla* R and S biotypes also produced comparable leaf areas and dry weights in this study. In this study, the greater retention in the biotype R contrasts greater resistance because a greater amount of herbicide is available to be absorbed. Therefore, retention capacity did not contribute to the higher imazamox resistance of the R biotype.

The exudation could contribute to the imazamox resistance in this biotype as an NTSR mechanism. Consistent with this idea, in the R biotype, lower ^14^C-imazamox contents were found in the root than in the untreated parts of the shoot. This result could be explained by the exudation of the herbicide by the root being more important in the R biotype. Moreover, large amounts of ^14^C-imazamox were found in the nutritive solution of both R and S plants. Accordingly, at 96 HAT via root application, the shoot content decreased, and the root content increased, with these changes being more intense in the R biotype. These results could be interpreted as a much higher retranslocation (upwards from roots to shoots and then downwards from shoots back to roots) in the R biotype, in agreement with results obtained in the assays with foliar herbicide application. Phosphorous images for both foliar and root applications also supported the hypothesis that imazamox could be eliminated from R plants through the roots. This is the first known case in which herbicide root exudation could contribute to imazamox resistance in *E. heterophylla*.

The only acknowledged previous case in which a similar NTSR mechanism has been described, that is, herbicide root exudation, was for an R biotype of *Raphanus raphanistrum* treated with MCPA, in which the herbicide was quickly translocated and high levels were exuded by the roots^33^. Herbicides can move inside the plant by diffusion, active transport and bulk transport. The third process is responsible for long-distance transport inside plants^52^. Considering the high rates of herbicide translocation and exudation by the roots, bulk transport was presumably responsible for imazamox movement. Differential herbicide transport between R and S plants can be attributed to ATP-dependent [ATP-binding cassette (ABC)] transporters^53^, which move the molecule into the vacuole or extracellular space^30,54–56^. Changes in the expression of NTSR genes, related to herbicide-metabolizing enzyme(s) or transporter proteins, can lead to an increase in herbicide degradation or translocation, respectively^57,58^.

It is not clear why this mechanism occurs and which phases are involved. However, it is known that the exudation of toxic compounds can be stimulated by abiotic and biotic stresses^59^. This is not the first time that the herbicide distribution and amount have been related to its effectiveness. In 1985, Turnbull and Stephenson^60^ began to relate the herbicide distribution and amount to the effectiveness of the herbicide, and in 2009, Bukun *et al*.^61^ proposed the biological activity of the herbicide as most important, followed by the amount and distribution. It could be hypothesized that this mechanism could eliminate high amounts of herbicide from the cell medium and contribute to resistance. Regardless of the chemical form (herbicide or metabolites) in which the herbicide is exuded, moving the substance outside of the plant (exudation) is synonymous with detoxification. In fact, herbicide effectiveness depends on the amount of herbicide that is able to reach the target protein in the plant^49^ On the other hand, considering that the enzyme is mutated in R plants, the herbicide could not bind to it and would remain free in tissues. This may allow enhanced translocation to other parts of the plant, including roots and exudation (supported by Supplementary Fig. S2). Therefore, it cannot be discarded that higher root exudation in the R biotype might be a side effect of the altered target site. To confirm that exudation contributes to resistance, non-mutated plants with root exudation and plants with only ALS mutations but no root exudation should be compared with this biotype. Unfortunately, these *E. heterophylla* biotypes do not exist yet, to the best of our knowledge.

This research described for the first time the resistance mechanisms to ALS inhibiting herbicides in this species. The principal mechanism that can explain the resistance in the R biotype is the mutation Ser653Asn (TSR). However, root exudation via the NTSR mechanism could also contribute to resistance by removing the toxic compound from the inside of the plant.

## Materials and Methods

### Plant material

Seeds of *E. heterophylla* from two biotypes, one resistant (R) and one susceptible (S) to ALS-inhibiting herbicides, were kindly provided by Dr. L. Vargas in 2015^12^ (area of Nova Boa Vista Li Perau, Brazil). One hundred seeds of each biotype were germinated and grown in 40 × 80 × 15 cm trays with a 2:1 (v/v) mixture of fertilized peat (COMPO SANA Universal, COMPO Ibérica, Spain) and sandy soil in a growth chamber at 28/18 °C (day/night) under a 16 hours photoperiod and an irradiance of 850 µmol m^−2^ s^−1^. When the plants had produced four fully extended leaves, they were treated with commercially formulated imazamox (Pulsar® 40, 4% w/v ai, BASF, Germany) at 40 g ai ha^−1^ in mixture with the adjuvant Dash® (34.5% w/v methyl oleate/methyl palmitate, BASF, Germany) at a dose of 1.25 L ha^−1^ using a treatment chamber (Devries Manufacturing, Hollandale, MN, USA) equipped with a TeeJet 8002 EVS flat fan nozzle calibrated to deliver 250 L ha^−1^ at 200 kPa at a height of 50 cm above plants. Only half of the plants from each biotype were treated with imazamox as described above. After 21 days, the R plants treated with imazamox exhibited 95% survival and no visual damage by the herbicide, while the S plants exhibited 100% mortality. The individuals of each biotype showed a high level of homogeneity in the response. Then, 20 R plants treated with the herbicide and 20 S plants not treated with herbicide were transplanted separately in the field (University of Córdoba) such that there was no possibility for cross pollination between them. When the plants reached maturity, seeds were collected and dried at 25 °C in the laboratory, labelled as being R or S to imazamox and later stored in a cold chamber at 4 °C. These F1 seeds were used in all subsequent trials.

### Growing conditions

Seeds were germinated in Petri dishes containing filter paper moistened with distilled water and placed in a growth chamber at 28/18 °C (day/night) with a photoperiod of 16 h, an irradiance of 850 µmol m^−2^ s^−1^ and a relative humidity of 80%. Seedlings were transplanted either into pots or into a hydroponic system, depending on the assay. Pots with a 500 mL volume contained fertilized peat (COMPO SANA Universal, COMPO Ibérica, Spain) and sand (2:1, v/v) as substrate and 1 plant/pot. In the hydroponic system, each plant was grown in an opaque container with 20 mL of continuously aerated nutrient solution. The nutrient solution had the following composition: 2 mM Ca(NO_3_)_2_, 0.75 mM K_2_SO_4_, 0.65 mM MgSO_4_, 0.5 mM KH_2_PO_4_, 50 μM KCl, 10 μM Fe-EDDHA, 10 μM H_3_BO_3_, 1 μM MnSO_4_, 0.5 μM CuSO_4_, 0.5 μM ZnSO_4_, and 0.05 μM (NH_4_)_6_Mo_7_O_24_. The pH was adjusted to 6.0 with 0.1 N KOH. All plants were grown in a growth chamber at 26/18 °C (day/night) under a 16 h photoperiod.

### Dose-response assays

*E. heterophylla* plants at the 4-leaf growth stage were treated with imazamox and the adjuvant as described above. The doses of imazamox for the S biotype were 0, 2.5, 5, 10, 20, 40, and 80 g ai ha^−1^, and those for the R biotype were 0, 25, 50, 100, 200, 400, 600, 1200 and 2400 g ai ha^−1^. The dose-response assays were performed using a randomized design with five repetitions per herbicide dose. Twenty-one days after treatment (DAT), the plants were cut at ground level and weighed to determine the fresh weight reduction (ED) and plant mortality (LD)^62,63^. A previous assay was performed to select the adequate dose range for both biotypes. The results of this assay were similar to those obtained in the present work (data not shown).

### ALS enzyme activity assays

The activity of the ALS enzyme was determined *in vitro* following the methodology from Shaner *et al*.^64^ and modified by Hatami *et al*.^65^ with two steps (extraction and enzymatic activity). To evaluate possible cross resistance patterns, the following ALS-inhibiting herbicides were tested: imazamox (imidazolinone, IMI), bensulfuron (sulfonylurea, SU), bispyribac (pyrimidinyl–thio–benzoate, PYR), florasulam (triazolopyridine, TRI) and flucarbazone (sulfonylamino-carbonyltriazolinone, SUCAR).

#### Extraction step

Samples of 3 g of young leaf tissue of each biotype from untreated plants at the 4-leaf growth stage were cut, identified, wrapped in aluminium foil and stored in liquid nitrogen. The samples were ground in a porcelain mortar using liquid nitrogen until a fine and homogeneous powder was obtained, to which 0.5 g of polyvinylpyrrolidone (PVPP) was added. The extraction buffer used at a ratio of 1:2 (g foliar tissue: mL buffer) was composed of 1 M K-phosphate buffer (pH of 7.5), 10 mM sodium pyruvate, 5 mM magnesium chloride, 50 mM thiamine pyrophosphate, 100 μM flavin adenine dinucleotide, 12 mM dithiothreitol, and 1:9 (v/v) glycerol:distilled water. The suspension was stirred for 10 min at 4 °C and subsequently filtered using four layers of cheesecloth. The filtered sample was centrifuged (15 min at 15,000 rpm and 4 °C). The supernatant was used immediately for enzymatic activity assays.

#### Enzymatic activity step

For *in vitro* bioassays of the enzyme, reactions were prepared in 2 mL Eppendorf tubes. Analytical-grade herbicides were used for this assay and purchased from Sigma Aldrich (St. Louis, MO USA). The herbicide concentrations used were 0, 0.1, 0.5, 1, 10, 100, 500, 1000 and 5000 µM. Twenty microliters of distilled H_2_O was used for the positive controls (100% ALS activity), and 50 μL of a solution of 1:50 (v/v) H_2_SO_4_-distilled H_2_O was added to the other Eppendorf tubes for the negative controls (0% ALS activity). Ninety microliters of the enzyme extract was added to each Eppendorf tube together with 110 μL of reaction buffer and herbicide concentrations. The mixture was incubated at 37 °C for 1 h, and the reaction was stopped by the addition of 50 μL of H_2_SO_4_ to the tubes, except for the negative control. A second incubation was performed at 60 °C for 15 min. To decarboxylate acetolactate in acetoin, 250 μL of creatine and 250 μL of naphthol were added. The tubes were again incubated for 15 min at 60 °C. The absorbance was measured at 520 nm with a spectrophotometer (mod. DU-640, Beckman, Fullerton, CA, USA). The total protein content was determined by the Bradford method^66^. The experiment was performed twice with three repetitions for each concentration of herbicide. The variance stability tests of enzymatic activity data (herbicide x concentration) showed no differences between replicates, and they were pooled within a single data set, i.e., the sample size was six repetitions per herbicide concentration.

### ALS sequencing

Leaf tissues (±100 mg per sample) of the R and S plants were used for DNA extraction with the Speedtools DNA Extraction Plant Kit (Biotools B and M Labs. S.A). The plants were selected by a previous herbicide treatment at a lethal dose. Only the surviving plants were selected. Two pairs of primers, ALS3B/ALS3F (5′-TCARTACTWAGTGCKACCATC-3′ and 5′-GGRGAAGCCATTCCTCC-3′, respectively) and P1/P2 (5′-GAAGCCCTCGARCGTCAAGG-3′ and P2 5′-ATAGGTTGWTCCCARTTAG-3′), were used to amplify fragments of 501 and 639 bp, respectively, of the CAD and BE domains of the ALS gene^67^. The polymerase chain reactions (PCRs) were performed with Certamp Complex Enzyme Mix (Biotools BandM Labs, Madrid, Spain) following the manufacturer’s instructions (50 ng of DNA, 0.2 mM of each primer, 1 µL of dNTPs at 10 mM each, 1 µL of 50 mM MgCl_2_, 2.5 µL of 10X buffer, 1 µL of Certamp complex enzyme mix, and sterile bidistilled water up to complete 25 µL the reaction). PCR conditions consisted of one denaturation cycle at 94 °C for 1 min, followed by 35 cycles of 94 °C for 1 min (denaturation), 52 °C for 30 s (annealing), and 72 °C for 1 min (extension step) and a final extension cycle (5 min at 75 °C)^67^. PCR product sizes were confirmed on 1% agarose gels by viewing them under UV light. Ten PCR products per biotype were sequenced by Sanger technology. ALS sequences were verified and assembled using SeqMan Pro 11 (DNASTAR, WI, USA) and Geneious 8.1.8 (Biomatters Ltd., Auckland, New Zealand) software, respectively. This study was performed in 10 plants of each biotype, with 2–3 PCR products per plant.

### Foliar retention of imazamox

For this experiment, the methodology described by Domínguez-Méndez *et al*.^23^ was followed. Imazamox was applied to potted plants at the 4-leaf growth stage under the conditions described in the dose-response assays. The solution contained 40 g ai ha^−1^ imazamox plus the adjuvant and 100 mg L^−1^ Na-fluorescein. After 30 min, once the treated leaves had dried, the shoots were cut and immediately placed into test tubes containing 50 mL of 5 mM NaOH and agitated for 30 seconds. The wash solution was recovered, and the fluorescein absorbance was measured in a spectrofluorometer at λ_exc_ of 490 nm and λ_emi_ of 510 nm. For the determination of dry matter, the shoots were placed in an oven at a temperature of 60 °C and dried for 72 h, and the weight of each sample was recorded. The retention was expressed in μL of herbicide per gram of dry matter. The experiment was conducted with a completely randomized delineation with seven repetitions per biotype and herbicide concentration.

### Absorption, translocation, root exudation and visualization of ^14^C-imazamox applied via foliage

**Figure 2:**
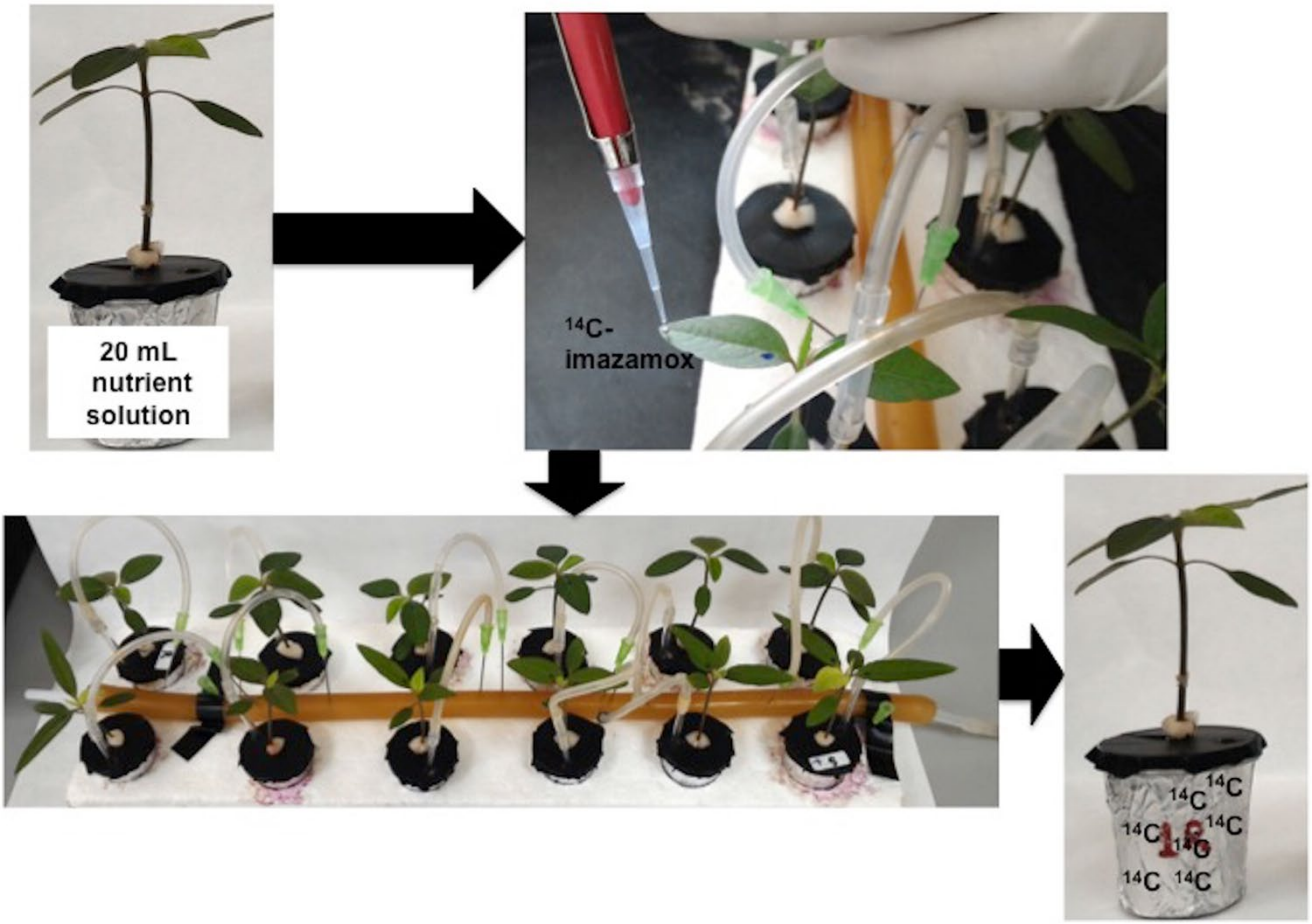
Design scheme used for the study of imazamox exudation in plants grown hydroponically.

Plants grown in a hydroponic system at the 4-leaf growth stage were treated with a solution of ^14^C-imazamox (1637 MBq/mmol) + commercially formulated imazamox + 1.25 L ha^−1^ adjuvant containing a specific activity of 1.67 kBq µL^−1^ (100,000 dpm) and an imazamox concentration of 40 g ai ha^−1^. One droplet (1 µL) of this solution was applied on the adaxial surface of a leaf of the second pair using a micropipette (LabMate) (Fig. 2). The time intervals studied were 3, 6, 12, 24, 48 and 96 hours after treatment (HAT). Three plants per biotype and evaluation time were used in a completely randomized design. At each sampled time, the treated leaf was washed twice with a water:methanol [10:90 v/v] solution to recover the unabsorbed ^14^C-herbicide. Each wash solution was mixed with 2 mL of scintillation liquid (Ultima Gold, Perkin-Elmer, BV BioScience Packard) and analysed by scintillation liquid spectrometry (LSS) (Scintillation counter, Beckman LS 6500). Whole-treated plants were carefully removed from the container and separated into the treated leaf, the rest of the shoot and the roots. Each sample was stored in a filter paper cone for combustion and dried at 60 °C for 72 h. The samples were individually combusted in a biological oxidizer (Packard Tri Carb 307, Packard Instrument Co., Downers Grove, IL, USA). The ^14^CO_2_ from the combustion was recovered in 18 mL of a mixture of Carbo-Sorb E and PermaFluor (1:1 v/v) (Perkin-Elmer, BV Bioscience Packard). The radioactivity was also quantified by LSS. The percentage of ^14^C recovered was estimated from the radioactive values as follows:

1 $$\begin{array}{c} \% {}_{{\rm{rec}}}{}^{14}\,{\rm{C}}=\frac{({\rm{kBq}}\,{\rm{treated}}\,{\rm{leaf}}+{\rm{kBq}}\,{\rm{rest}}\,{\rm{of}}\,{\rm{shoot}}+{\rm{kBq}}\,{\rm{roots}}+{\rm{kBq}}\,{\rm{from}}\,{\rm{washes}}+{\rm{kBq}}\,{\rm{from}}\,{\rm{nutrient}}\,{\rm{solution}})}{{\rm{total}}\,{\rm{kBq}}\,{\rm{applied}}}\times 100\end{array}$$

The ^14^C translocation was visualized in another group of plants using a phosphor imager (Cyclon, Perkin-Elmer, Packard Bioscience BV). Whole plants, treated as described previously, were gently dried and fixed on filter paper (25 × 12.5 cm) at room temperature. Subsequently, the plants were placed on phosphor storage film (Storage Phosphor System: Cyclone, Perkin–Elmer Packard BioScience BV) for a period of 4 h to determine the distribution of the radiolabelled herbicide. Three plants were used per biotype and time to observe the translocation of ^14^C. In addition, to quantify the ^14^C exuded via the root system, 20 mL of nutrient solution was added, and then 1 mL was taken, mixed with 2 mL of scintillation liquid and quantified by LSS.

### Imazamox metabolism

At the 4-leaf stage, plants were treated with the herbicide via foliage as described for the dose-response assays but grown in a hydroponic system. In this case, the dose was 40 g ai^−1^ ha^−1^ imazamox + 1.25 L ha^−1^ adjuvant. Plants were collected at 6, 12, 24, 48, 96 and 168 HAT. There were three plants per biotype and sampled time. The following methodology was described by Rojano *et al*.^32^. At each time point, plants were extracted from the container and divided into aerial parts and roots, and the nutrient solution was also conserved. The aerial part and the root were individually ground into powder in a porcelain mortar using liquid nitrogen. The samples were transferred to recipient tubes with 5 mL of methanol/water [95:5 (v/v)] and then sonicated at 70 W for 15 min with a duty cycle of 70% (0.7 s/s). The extract was isolated by centrifugation (15 min at 12,000 rpm). The resulting solid was subjected to extraction two more times, using 5 mL of methanol/water [95:5 (v/v)] each time. After obtaining 15 mL of solution, the samples were evaporated to dryness under a stream of nitrogen. The solid residue was reconstituted in 0.5 mL of methanol/water [95:5 (v/v)] and filtered using a syringe (2.5 mL) through a nylon filter (45 μm pore size and 13 mm internal diameter). For the nutrient solution, all the mixture in the container was evaporated and then reconstituted in 0.5 mL of methanol/water.

The samples were analysed using a 15 Gold HPLC System from Beckman Coulter (Fullerton, USA) equipped with a 26 System Gold Diode Array detector (wavelength range of 190–600 nm). A hydrophilic interaction liquid chromatography column (20 × 4.6 cm, 3 μm particle size) was used to separate the desired compounds. Fifty microliters of the reconstituted sample was injected into the liquid chromatograph, with 1% (v/v) acetic acid in water as mobile phase A and pure methanol as mobile phase B. Imazamox and its metabolites were determined by LC-UV absorption analysis at a wavelength of 240 nm. The elution program started with 5% mobile phase B, followed by a linear gradient of step 1 (5% to 20% methanol for 10 min), step 2 (20% to 80% methanol for 10 minutes), step 3 (80% to 100% methanol for 5 minutes) and step 4 (100% to 5% methanol for 10 minutes). The temperature and constant flow rate of the column were 40 °C and 1.0 mL min^−1^, respectively. The quantification of imazamox metabolites was based on the calibration model prepared with commercial imazamox. The chromatographic peaks represented in LC-UV were assigned in accordance with the retention times.

### Accumulation, distribution and visualization of ^14^C-imazamox applied to the roots

Plants were grown under the hydroponic system described above, and when they were at the 4-leaf growth stage, 1 µL of the previous radiolabelled solution (1.67 kBq μL^−1^, 100,000 dpm, imazamox concentration of 40 g ai ha^−1^) was added to the nutrient solution (20 mL). At 24 and 96 HAT, the plants were carefully removed from the container. The roots were washed with deionized water, and the plants were separated into shoots and roots. Three plants per time and biotype were used. Plant tissue samples, washes and nutrient solutions were prepared to quantify their radioactivity by LSS as in the previous section. The recovery was estimated from the radioactive values as follows:

2 $${\boldsymbol{ \% }}\,{}_{{\boldsymbol{rec}}}{}^{14}{\boldsymbol{C}}=\frac{({\rm{kBq}}\,{\rm{shoot}}+{\rm{kBq}}\,{\rm{roots}}+{\rm{kBq}}\,{\rm{from}}\,{\rm{nutrient}}\,\mathrm{solution}\,)}{{\rm{total}}\,{\rm{kBq}}\,{\rm{applied}}}\times 100$$

The root translocation of imazamox was also visualized using a phosphor imager (Cyclon, Perkin-Elmer, Packard Bioscience BV) as described for the assays with foliar application, using another group of three plants per biotype and time.

### Data analysis

Outliers of percentage data of the response-dose assays (whole plants and ALS enzymatic activity) were identified and removed based on the internally studentized residual method with α = 0.05. Then, the amount of herbicide causing a reduction in fresh weight compared to the untreated control (ED_50_), mortality (LD_50_) or ALS activity reduction by 50% (I_50_) was calculated by submitting the percentage data to a non-linear regression analysis using a logistic model of three parameters (four parameters (Y = c + {(d − c)/[1 + (x/g)^b^]}, where *c* and *d* are the upper and lower asymptotic limits, *b* is the slope, *g* is the ED_50_ or I_50_, and x is the herbicide concentration)^63^. Regression analyses were performed in SigmaPlot 10.0 software (Systat Software Inc.) with the R program. The resistance factor (RF) was calculated as FR = R/S by using the corresponding ED_50_, LD_50_ or I_50_ values of the R and S *E. heterophylla* biotypes.

Negative control values of the enzymatic activity were submitted to analysis of variance (ANOVA) to search for differences in the initial concentration of the ALS (basal activity) between both biotypes in a completely randomized scheme (2 × 6). Data from foliar retention were submitted to ANOVA in a completely randomized design. Data from the foliar absorption and translocation and from accumulation and distribution applied to the roots (for each plant section) assays, as well as metabolism of ^14^C-imazamox, were analysed as factorial schemes (biotypes by time points) using ANOVA. Model assumptions of normal distribution of errors and homogeneous variance were graphically inspected for all tests. Values of P < 0.05 were considered statistically significant and multiple mean comparisons were performed using the Tukey’s test at the 5%. Statistical analyses were conducted with the Statistix 9.0 software (Analytical Software, Tallahassee) for basal activity, foliar retention and absorption and translocation assays. The STATGRAPHICS Plus program (v 4.0) was used for data processing of imazamox metabolism.

## Supplementary information

Supplementary Figures
