# Supplementary material for: Target site as the main mechanism of resistance to imazamox in a Euphorbia heterophylla biotype
Source: Sci Rep. 2019 Oct 28;9:15423. doi: 10.1038/s41598-019-51682-z (PMC6817884; doi:10.1038/s41598-019-51682-z)
Supplement: Supplementary file 1 — Supplementary Figures [file 41598_2019_51682_MOESM1_ESM.pdf]

Target site as the main mechanism of resistance to imazamox in a *Euphorbia heterophylla* biotype

Antonia M. Rojano-Delgado[1*], João M. Portugal[2], Candelario Palma-Bautista[1], Ricardo Alcántara de la Cruz[3], Joel Torra[4], Esteban Alcántara[5], Rafael De Prado[1]

[1]Department of Agricultural Chemistry and Edaphology, University of Córdoba, Córdoba, Spain

[2]Department of Biosciences, Research Center for Endogenous Resource Valorization's, Polytechnic Institute of Beja, Beja, Portugal

[3] Department of Chemistry, Federal University of São Carlos, São Carlos, Brazil.

[4]Department d'Hortofructicultura, Botànica i Jardineria, Agrotecnio, Universitat de Lleida, Lleida, Spain

[5]Department of Agronomy, University of Córdoba, Córdoba, Spain

*** Corresponding author:** Antonia M. Rojano-Delgado q92rodea@uco.es

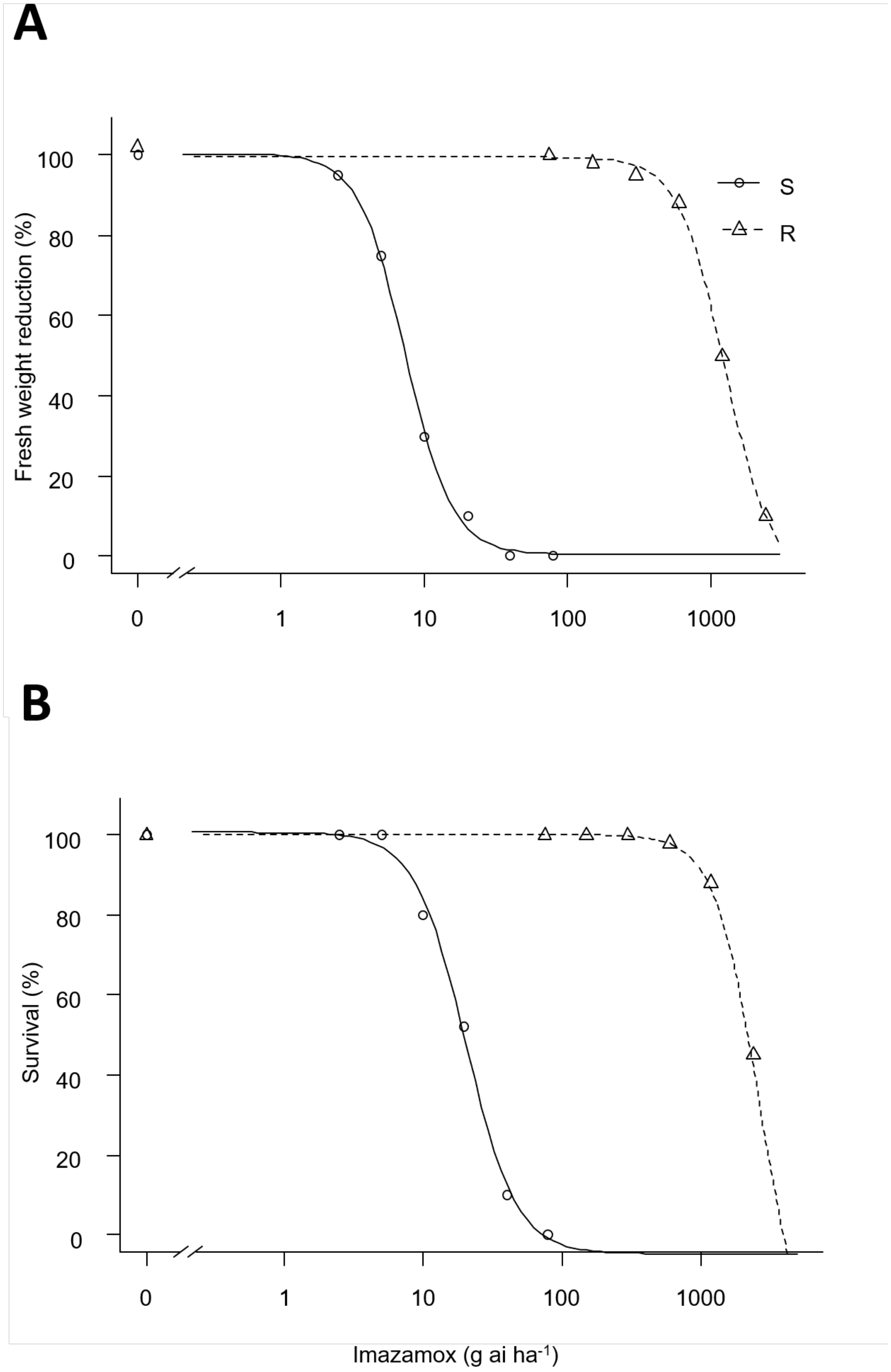

Supplementary Fig. S1. Log–logistic curves of imazamox-susceptible and -resistant *E. heterophylla* biotypes evaluated at 21 DAT. (A) Dose–response curves with respect to percentage of fresh weight reduction. (B) Dose–response curves with respect to percentage of survival.

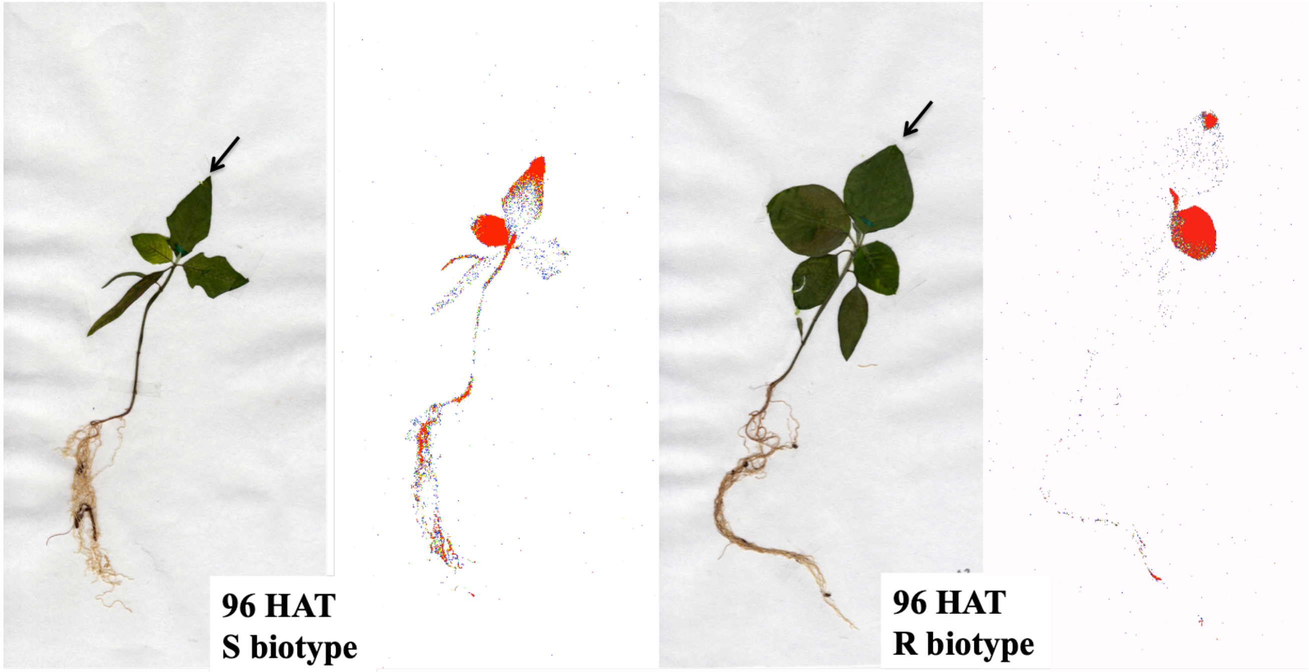

Supplementary Fig. S2. Representative images at 96 HAT with foliar application demonstrating the movement of ^{14}C-imazamox in plants of the S (left) and R (right) biotypes *of E. heterophylla*. A greater intensity of the red color indicates a higher concentration of ^{14}C-imazamox. The arrows indicate the treated leaves.

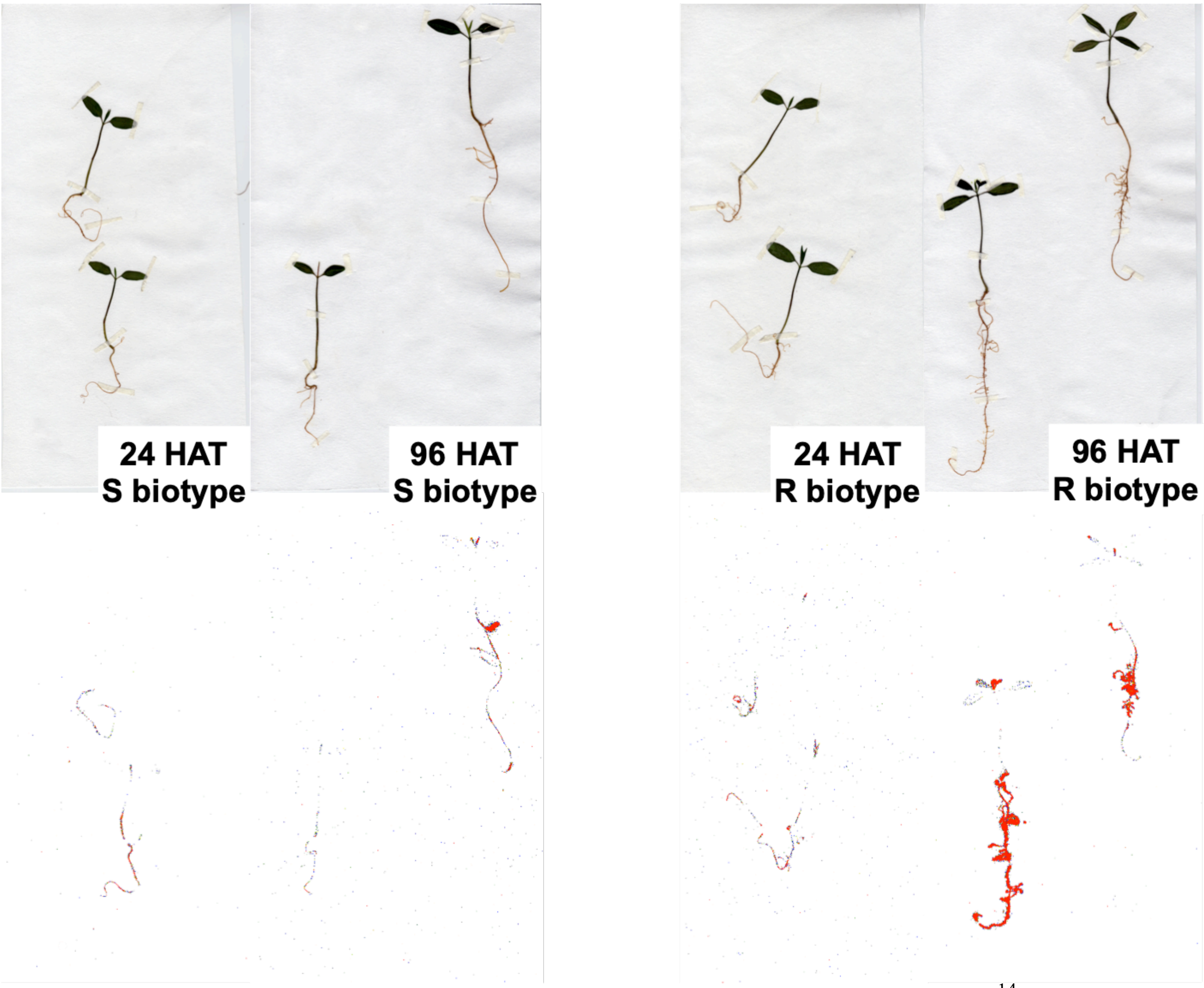

Supplementary Fig. S3. Representative images demonstrating the movement of ^{14}C-imazamox in plants of the S (left) and R (right) biotypes *of E. heterophylla*. The images were recorded at different times in the root-application assay. A greater intensity of the red color indicates a higher concentration of ^{14}C-imazamox
